# Real‐World Patient Eligibility for Alzheimer's Disease Anti‐Amyloid Therapy in Seven Swedish Memory Clinics

**DOI:** 10.1111/ene.70786

**Published:** 2026-10-20

**Authors:** Anna Rosenberg, Anna Rennie, Urban Ekman, Makrina Daniilidou, Anette Hall, Anna Matton, Ari Solomon, Lars‐Olof Wahlund, Miia Kivipelto, Eric Westman

**Affiliations:** ^1^ Division of Clinical Geriatrics, Department of Neurobiology, Care Sciences and Society Karolinska Institutet Stockholm Sweden; ^2^ Institute of Public Health and Clinical Nutrition University of Eastern Finland Kuopio Finland; ^3^ Women's Health and Allied Health Professionals Theme Karolinska University Hospital Stockholm Sweden; ^4^ FINGERS Brain Health Institute Stockholm Sweden; ^5^ Department of Neurology, Institute of Clinical Medicine University of Eastern Finland Kuopio Finland; ^6^ Ageing Epidemiology (AGE) Research Unit, School of Public Health Imperial College London London UK; ^7^ Theme Inflammation and Aging Karolinska University Hospital Stockholm Sweden

**Keywords:** Alzheimer's disease, anti‐amyloid therapy, donanemab, eligibility, lecanemab

## Abstract

**Background:**

Anti‐amyloid therapies lecanemab and donanemab are the first potentially disease‐modifying treatments for Alzheimer's disease (AD) approved in Europe. We estimated real‐world eligibility for these treatments in a large multicenter memory clinic patient cohort in Sweden (MemClin).

**Methods:**

MemClin included patients with a first diagnostic visit between 2016 and 2022 at seven specialist memory clinics in Stockholm, Sweden. To estimate eligibility for anti‐amyloid therapy, we applied key inclusion/exclusion criteria from the lecanemab and donanemab appropriate use recommendations. Eligible patients were required to have (1) a clinical diagnosis of AD‐type dementia or mild cognitive impairment (MCI), (2) confirmed Aβ pathology (abnormal CSF Aβ42) and (3) no apparent medical exclusion criteria. Eligibility was calculated for different scenarios to provide both upper‐bound estimates (main analysis) and more conservative, implementation‐ready estimates of near‐term treatable patients (sensitivity analysis).

**Results:**

Cohort consisted of 2126 patients (mean age 77.1 ± 6.6 years; 61.8% MCI, 24.2% dementia, 13.7% subjective cognitive impairment). In the main analysis, 11.5% of the whole cohort was estimated to be eligible for either treatment (lecanemab 10.6%, donanemab 11.5%). Most eligible patients had early‐stage disease (61.9% MCI vs. 38.1% dementia) and an A + T– biomarker profile, that is, normal biomarker of tau (57.9%). Eligibility was markedly lower in sensitivity analyses (e.g., 2.7% when all patients without MRI were assumed ineligible).

**Conclusions:**

Even upper‐bound estimates indicate that a minority of real‐world memory clinic patients may currently qualify for anti‐amyloid therapy. These findings provide timely evidence to inform the ongoing debate on the implementation of anti‐amyloid therapy in public healthcare.

## Graphical abstract

Anti‐amyloid therapies lecanemab and donanemab are targeted at patients with early Alzheimer‐type dementia or mild cognitive impairment (MCI). In a large, Swedish multicenter memory clinic patient cohort, we found an eligibility rate of 11.5% for either treatment in the whole cohort (18.1% among dementia patients, 11.5% among aMCI patients), indicating that only a minority of real‐world memory clinic patients may currently qualify for anti‐amyloid therapy. Our findings inform the ongoing debate on the implementation of anti‐amyloid therapy in public healthcare.

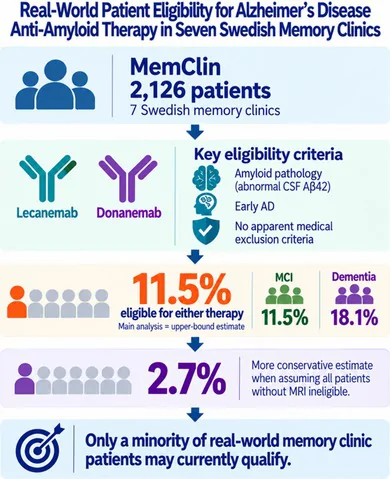

**Abbreviations**
AD: Alzheimer's disease
APOE: apolipoprotein E
ARIA: amyloid‐related imaging abnormalities
ATN: amyloid, tau, neurodegeneration biomarker classification system
AUR: appropriate use recommendations
Aβ: amyloid‐β
CSF: cerebrospinal fluid
CT: computed tomography
DMT: disease‐modifying therapy
DSM‐V: Diagnostic and statistical manual of mental disorders, fifth edition
GCA: global cortical atrophy
ICD‐10: International classification of diseases, tenth revision
MCI: mild cognitive impairment
MMSE: Mini‐Mental State Examination
MoCA: Montreal Cognitive Assessment
MRI: magnetic resonance imaging
MTA: medial temporal lobe atrophy
NFL: neurofilament light chain
PET: positron emission tomography
p‐tau: phosphorylated tau
SCI: subjective cognitive impairment
t‐tau: total tau

## 1 Introduction

The approval of monoclonal anti‐β‐amyloid (Aβ) antibodies lecanemab (Leqembi) and donanemab (Kisunla), has reshaped the therapeutic landscape of Alzheimer's disease (AD). In Phase 3 clinical trials, both treatments slowed cognitive and functional decline and cleared brain Aβ, with effects also on cerebrospinal fluid (CSF) and blood biomarkers of tau pathology [1, 2]. Lecanemab and donanemab have received regulatory approval in several countries including for example, the United States, the United Kingdom, China, South Korea, Japan, Australia, and European Union [3]. They are indicated for patients with mild cognitive impairment (MCI) or mild dementia due to AD, and require biomarker confirmation of Aβ pathology (currently via CSF or amyloid‐PET). Exclusion criteria are for example, substantial vascular co‐pathology, existing brain hemorrhages, certain comorbidities, and anticoagulant use, all of which increase the risk of amyloid‐related imaging abnormalities (ARIA) [4, 5, 6]. In Europe, for example, also apolipoprotein E (*APOE*) ε4/4 carriers are excluded due to increased ARIA risk [6, 7, 8].

Although AD remains an area of high unmet medical need, the widespread rollout of lecanemab and donanemab is currently pending in Europe. Some regulatory agencies, for example, in Sweden, France, and the UK, have advised against the use of anti‐amyloid therapy in public healthcare due to modest benefits, safety concerns, and/or limited cost‐effectiveness [9, 10, 11]. Implementation of anti‐amyloid therapy poses a challenge to healthcare systems as it requires redefining and developing the diagnostic pathways and infrastructure for patient monitoring [12, 13, 14]. First experiences in Europe nevertheless highlight that it can be feasible [13]. To inform the decision‐making about the implementation of anti‐amyloid therapy, it is important to quantify the near‐term real‐world volume of treatment‐eligible patients. In Europe, an estimated 22 million individuals have symptomatic AD (MCI or dementia due to AD) [15], and in Sweden approximately 100,000 individuals are living with AD [11]. While these figures highlight the scale of potential demand for disease‐modifying therapy, not all patients will qualify for treatment, and more nuanced analyses of real‐world eligibility rates are needed. Estimating eligibility is particularly relevant in settings where patients with cognitive concerns are currently typically evaluated, and where anti‐amyloid therapy will likely first be rolled out (specialist memory clinics) [12].

In this study, we estimated eligibility for anti‐amyloid therapy in a real‐world memory clinic setting, leveraging the published clinical appropriate use recommendations (AUR) for lecanemab and donanemab [4, 5]. Expanding on our prior work from a single memory clinic [16, 17], we analyzed here the large, more representative MemClin cohort, which covers seven different outpatient memory clinics in Stockholm, Sweden.

## 2 Methods

### 2.1 Study Setting and Participants

This study was conducted as part of the Swedish multi‐center memory clinic study, MemClin. MemClin coordinates data collection across memory outpatient clinics in Stockholm, Sweden, with the aim to create a harmonized, large‐scale database to improve diagnostics and care of dementia disorders. MemClin began in 2016 and included new patients until 2022. Details of the MemClin study design and methods are available elsewhere [18].

Eight out of ten memory clinics in Stockholm participate in MemClin, serving individuals with cognitive concerns from Stockholm County and surrounding suburban areas (catchment area ~2.1 million of the total 2.3 million inhabitants; information from statistics Sweden, 2020 [18]). Patients are typically referred to memory clinics by general practitioners in primary care when a basic dementia examination does not yield a clear diagnosis. This evaluation includes medical history and clinical status, structured interview with relatives, blood tests, head MRI/CT (CT often the first‐line modality due to availability), and cognitive screening (Mini‐Mental State Examination, MMSE, and clock‐drawing tests). Major medical conditions (e.g., cardiovascular disease, depression, cancer) are typically required to be stabilized before referral to the memory clinics, or at least before the referral is accepted.

Diagnostic assessments at the memory clinics follow the Swedish National Board of Health and Welfare guidelines [19]. The diagnostic workflow includes for example, comprehensive medical and neurological examinations, informant‐based history, blood chemistry, and neuropsychological testing (covering episodic memory, executive functioning, attention/working memory, visuospatial functions, language, and semantic memory domains; with some variation in tests across clinics). AD biomarkers are measured in CSF for a subset of patients, particularly when AD is suspected. CSF biomarker analysis is the primary method to assess AD pathology (amyloid‐PET is not part of routine clinical workflow). However, lumbar puncture can be omitted from routine diagnostic assessments for example, due to contraindications, excessive patient burden, or when the suspected diagnosis is not AD [19].

The diagnostic procedures at the memory clinics are guided by clinical judgment (responsible geriatricians decide the necessary examinations), and data availability therefore varies among patients. Final diagnoses are determined through consensus at each clinic following the harmonized diagnostic criteria (DSM‐V and ICD‐10 coding, in conjunction with the consensus diagnostic criteria for MCI [20]). Patients without objective cognitive decline are considered to have subjective cognitive impairment (SCI).

MemClin leverages routinely collected patient data, meaning that it did not interfere with clinical workflows or include any additional research‐related assessments. The study design is naturalistic, with the only inclusion criterion being a neuropsychological evaluation, as neuropsychologists collected the written informed consents. All participants provided an informed consent, and the study has ethical approval (Regional Ethical Review Board in Sweden; Dnr: 2016/29‐31/1).

For this study, we included data from the first diagnostic visit of all MemClin patients, excluding those examined at the Karolinska University Hospital Solna memory clinic (final sample *N* = 2126, from seven memory clinics). Eligibility for anti‐amyloid therapy in the Solna clinic was investigated in our previous work [16, 17]. This clinic has a distinct responsibility profile and a fast‐track assessment model, resulting in a significantly younger patient population (mean age ~59 years). Also, unlike in most memory clinics, AD biomarker assessments and *APOE* genotyping are part of the routine diagnostic workup in Solna regardless of the suspected cause of cognitive symptoms [16, 17].

### 2.2 Biomarker Assessments and ATN Biomarker Classification

Neuroimaging data was derived from different imaging centers in Stockholm. Patients had either a high‐resolution head CT or/and an MRI (with a 1.5 T or 3 T scanner) including a high resolution sagittal T1‐weighted sequence for structural imaging (integrity of gray and white matter structures), a T2‐weighted sequence for additional contrast between CSF and tissue to exclude other medical causes, and a fluid‐attenuated inversion recovery (FLAIR) sequence [18]. Visual assessments included ratings for white matter lesions (Fazekas scale, 0–3), medial temporal lobe atrophy (MTA, Scheltens scale, 0–4), and global cortical atrophy (GCA, 0–3) [21, 22, 23, 24]. The scans and ratings were performed as part of the routine clinical workflow, and scores were extracted from the neuroradiology reports. Assessment of inter‐rater reliability was not performed.

CSF AD biomarker analysis included Aβ42, Aβ42/40, phosphorylated tau 181 (*p*‐tau_181_), total tau (*t*‐tau), and neurofilament light (NFL), with assay methods varying by clinic (ELISA, ECLIA, or CLEIA). The cut‐offs for abnormality provided by the laboratory/manufacturer were the following: for Aβ42 ≤ 550 ng/L (ELISA), ≤ 599 ng/L (ECLIA), and ≤ 620 ng/L (CLEIA); for *p*‐tau_181_ ≥ 80 ng/L (ELISA), ≥ 56.5 ng/L (ECLIA), and ≥ 61 ng/L (CLEIA); for total tau ≥ 400 ng/L (ELISA), ≥ 404 ng/L (ECLIA), and ≥ 360 ng/L (age < 50 years) and ≥ 479 ng/L (age 50+ years) (CLEIA); and for NFL ≥ 380 ng/L (age < 30 years), ≥ 560 ng/L (age 30–39 years), ≥ 890 ng/L (age 40–59 years), and ≥ 1850 ng/L (age 60+ years).

We mapped patients' biomarker profiles according to the Amyloid‐Tau‐Neurodegeneration (ATN) framework which classifies individuals based on AD biomarkers regardless of the cognitive status or clinical disease stage [25]. It defines eight biomarker profiles based on the presence (+) or absence (−) of pathological markers for Aβ (A), tau (T), and neurodegeneration (N). Profiles fall under three main categories: normal profile (A – T −  N−), non‐AD pathologic change (A – T +  N±, A – T –  N+), and Alzheimer's continuum (A + T ±  N±). Aligned with our previous work [16], we based the classification on CSF Aβ42 (A), CSF p‐tau_181_ (T), and MTA (N). We defined abnormal CSF biomarkers using the standard method‐specific cut‐offs provided by the laboratory/manufacturer, and abnormal MTA based on previously published age‐specific thresholds: ≥ 1.5 for patients < 75 years, ≥ 2 for patients aged 75–84 years, and ≥ 2.5 for patients 85+ years [26].

### 2.3 Eligibility for Anti‐Amyloid Therapy

To estimate the number of patients potentially eligible for anti‐amyloid therapy, we applied inclusion and exclusion criteria outlined in the clinical appropriate use recommendations (AUR) for lecanemab and donanemab [4, 5], along with the current European recommendations and regulatory label guidelines regarding brain amyloid pathology [7, 8, 27]. Eligibility criteria in the AURs and their exact operationalization in this study are detailed in Tables S1 and S2.

Briefly, patients were considered eligible if they met the following criteria:

1. Performed lumbar puncture to assess CSF AD biomarkers and confirmed Aβ pathology, defined as abnormal CSF Aβ42 based on the standard laboratory cut‐offs used in routine clinical practice (different cut‐offs for the different assays). Evidence of abnormal Aβ, regardless of tau, is the current European recommendation in the regulatory label guidelines.
2. Clinical diagnosis of AD‐type dementia or MCI, with no indication of a non‐AD neurological disorder. Patients with substantial cognitive impairment MMSE < 22 or Montreal Cognitive Assessment (MoCA) < 17 (lecanemab), or MMSE < 20 or MoCA < 13 (donanemab), were excluded.
3. No apparent medical exclusion criteria, for example, documented anticoagulant use, severe comorbidity contraindicating treatment, or severe white matter hyperintensities (Fazekas rating 3) on MRI or CT. No other neuroimaging‐related exclusion criteria could be applied in this study, such as those related to micro‐ and macrohemorrhages, superficial siderosis, and imaging patterns suggestive of cerebral amyloid angiopathy (Tables S1 and S2). Also, not all exclusion criteria related to patient medical history/comorbidities could be confirmed (e.g., prior seizures, detailed immunologic comorbidity/immunosuppressants; Tables S1 and S2).

CT is currently the standard imaging modality in primary care, and most patients in the MemClin cohort underwent CT instead of MRI. As the absence of MRI did not necessarily indicate contraindications, we did not automatically exclude patients without an MRI in the main eligibility analysis. This analysis yielded upper‐bound estimates of eligibility. Given that MRI is currently mandatory for both baseline safety screening and ARIA monitoring when anti‐amyloid therapy is considered, we performed an additional scenario analysis where we assumed lack of MRI to equal ineligibility until MRI is completed. While likely an overly conservative approach, this was done to provide more implementation‐ready estimates of near‐term treatable patients.

We also performed a sensitivity analysis to estimate how many patients would remain eligible for lecanemab or donanemab after excluding *APOE* ε4/4 carriers, which is currently a regulatory requirement in Europe. *APOE* data were not available in the MemClin cohort, as genotyping is currently not routine clinical practice in memory clinics [19]. We therefore applied published estimates of the proportion of ε4/4 carriers among otherwise treatment‐eligible patients from external cohorts to MemClin. These included the Karolinska University Hospital Solna memory clinic cohort in Sweden [17], a Dutch memory clinic cohort [28], and the Swedish population‐based Gothenburg H70 Birth Cohort [29]. In the most permissive scenario, we assumed that 13% of otherwise eligible MemClin patients would be ε4/4 carriers, based on the population‐based study [29]. The more restrictive scenarios assumed a prevalence of 26%, based on the Dutch memory clinic study [28], and 46%, based on the Solna memory clinic study [17].

### 2.4 Statistical Analysis

Group differences in patient characteristics were assessed with the chi‐square test, *t*‐test, one‐way ANOVA, and Kruskal‐Wallis tests, as applicable. The threshold of significance was set at *p* < 0.05. Analyses were performed with Stata, version 18.

## 3 Results

### 3.1 Study Population Characteristics

Characteristics of the whole MemClin patient population, overall and per diagnostic group, are summarized in Table 1. Patients were on average 77.1 (SD 6.6) years old (range 39–94 years) and 50.4% were women. Most patients had MCI (61.8%, *n* = 1313); 13.7% (*n* = 291) had SCI and 24.2% (*n* = 514) had dementia. As for comorbidities, for example, 50.5% of all patients in this cohort had hypertension, 14.6% had diabetes, and 16.6% used anticoagulation treatment (Table 1). AD was the most common type of dementia (*n* = 356; pure AD *n* = 206, 40.1% of all dementia cases and mixed AD/vascular dementia *n* = 150, 29.2% of all dementia cases).

**TABLE 1:** Demographics, cognition and clinical characteristics of the study cohort.

Characteristic	*n* with data	All *N* = 2126	SCI *n* = 291	MCI *n* = 1313	Dementia *n* = 514	*p*
*Demographics*						
Age, years	2126	77.1 (6.6), range 39–94	74.7 (7.1)	77.1 (6.5)	78.2 (6.5)	**< 0.001**
Women	2126	1071 (50.4%)	174 (59.8%)	639 (48.7%)	254 (49.4%)	**0.002**
Education, years	1805	12.7 (3.7)	13.6 (3.7)	12.8 (3.6)	12.1 (3.7)	**< 0.001**
*Cognition*						
MMSE	1989	27.0 (2.4)	28.8 (1.5)	27.3 (2.0)	25.3 (2.6)	**< 0.001**
MoCA	97	24.7 (3.1)	27.1 (2.1)	25.3 (2.4)	21.9 (2.9)	**< 0.001**
RAVLT learning	1406	31.6 (11.0)	45.3 (9.1)	31.3 (9.6)	24.1 (7.5)	**< 0.001**
RAVLT delayed recall (verbal episodic memory)	1015	4.8 (3.8)	9.9 (2.8)	4.8 (3.4)	2.4 (2.6)	**< 0.001**
RCF copying	1452	28.8 (5.7)	32.3 (2.7)	28.8 (5.3)	26.5 (7.2)	**< 0.001**
RCF immediate recall (non‐verbal episodic memory)	1350	9.4 (6.8)	16.2 (6.3)	9.2 (6.2)	5.1 (4.9)	**< 0.001**
WAIS‐IV Digit span (working memory)	1441	21.3 (4.7)	24.6 (4.2)	21.3 (4.5)	19.4 (4.2)	**< 0.001**
Verbal fluency, FAS (executive functioning)	1517	34.4 (14.1)	44.0 (13.5)	34.8 (13.5)	27.9 (12.7)	**< 0.001**
WAIS‐IV Block design (visuospatial functions)	1365	27.6 (9.7)	36.1 (9.4)	27.8 (8.7)	22.8 (8.9)	**< 0.001**
WAIS‐IV Information (semantic memory)	1608	13.6 (4.9)	16.6 (3.8)	13.8 (4.8)	11.5 (4.7)	**< 0.001**
*Clinical characteristics, comorbidity, medical history*						
BMI, kg/m^2^	1712	25.4 (4.0)	25.2 (3.7)	25.6 (4.1)	24.9 (4.0)	0.05
History of stroke/TIA	2126	350 (16.5%)	32 (11.0%)	232 (17.7%)	86 (16.7%)	**0.02**
History of myocardial infarction	2126	128 (6.0%)	7 (2.4%)	82 (6.2%)	39 (7.6%)	**0.01**
Atrial fibrillation	2126	323 (15.2%)	25 (8.6%)	211 (16.1%)	86 (16.7%)	**0.003**
Anticoagulation treatment	2126	353 (16.6%)	34 (11.7%)	230 (17.5%)	89 (17.3%)	0.24
Hypertension diagnosis	2126	1074 (50.5%)	122 (41.9%)	676 (51.5%)	274 (53.3%)	**0.01**
Antihypertensive use	2126	870 (40.9%)	96 (33.0%)	547 (41.7%)	223 (43.4%)	**0.01**
Hyperlipidemia diagnosis	2126	341 (16.0%)	37 (12.7%)	209 (15.9%)	95 (18.5%)	0.10
Diabetes diagnosis (any type)	2126	310 (14.6%)	30 (10.3%)	192 (14.6%)	87 (16.9%)	**0.04**
Depression diagnosis	2126	217 (10.2%)	31 (10.7%)	141 (10.7%)	45 (8.8%)	0.44

*Note:* Data are shown as mean (SD) and *n* (%). *n* = 8 with a clinical diagnosis other than SCI, MCI, or dementia. Cognitive test results are expressed as raw scores. *p*‐values marked in bold are statistically significant < 0.05. The prevalence of hypertension and anticoagulant use is broadly consistent with for example, previous reports from the SveDem, Sweden's national quality registry for dementia disorders (Cermakova et al. *Journal of Alzheimer's Disease*. 2015), and other reports from memory clinic populations with high mean age like in MemClin (e.g., Defrancesco et al. *BJPsych Open*. 2024).

Abbreviations: BMI, body mass index; FAS, verbal fluency test with letters F,A,S; MCI, mild cognitive impairment; MMSE, mini‐mental state examination (range 0–30); MoCA, montreal cognitive assessment (range 0–30); RAVLT, Rey auditory verbal learning test; RCF, Rey complex figure test; SCI, subjective cognitive impairment; TIA, transient ischemic attack; WAIS‐IV, Wechsler adult intelligence scale, fourth edition.

CSF biomarker and neuroimaging data, and patient AD biomarker profiles (according to the ATN classification) are shown in Table S3 and Figure S1. CSF biomarkers were analyzed in 43.2% (*n* = 918) of all patients in this cohort, and 34.3% of them had abnormal Aβ42. Compared to patients who were analyzed for CSF biomarkers, patients who did not undergo a lumbar puncture to collect CSF were older and more likely to, for example, use anticoagulants and have comorbidities that may contraindicate the procedure (Table S4). Of all patients with imaging (*n* = 2060), 74.0% had a CT scan and 26.0% had an MRI. The median MTA rating was 1 (IQR 1–2), Fazekas score 1 (IQR 0.5–2), and GCA score 1 (IQR 1–2) (Table S3). The most common of the eight ATN biomarker profiles in this cohort was A − T − N − (the normal profile, 29.3%), and the least common profile was A + T + N + (5.4%; Figure S1A).

### 3.2 Eligibility for Anti‐Amyloid Therapy

#### 3.2.1 Main Analysis

A detailed breakdown of the eligibility assessment and numbers of patients assumed ineligible at each step of the assessment are shown in Figure 1 (lecanemab) and Figure 2 (donanemab). In total, 225 patients (10.6% of the whole cohort) met the eligibility criteria for lecanemab (Figure 1), and 244 patients (11.5% of the whole cohort) met the eligibility criteria for donanemab (Figure 2). The groups mostly overlapped due to the similar eligibility criteria (*n* = 225 potentially eligible for both treatments, *n* = 19 for donanemab only). Altogether, 244 patients (11.5% of the whole cohort) were considered potentially eligible for either treatment. This corresponds to 11.5% of all patients with MCI (151/1313) and 18.1% of all patients with any dementia (93/514). Among all patients with AD‐type dementia, the eligibility rate was 26.1% (93/356). Figures S2–S5 show the eligibility assessment separately for MCI and dementia patients.

**FIGURE 1:**
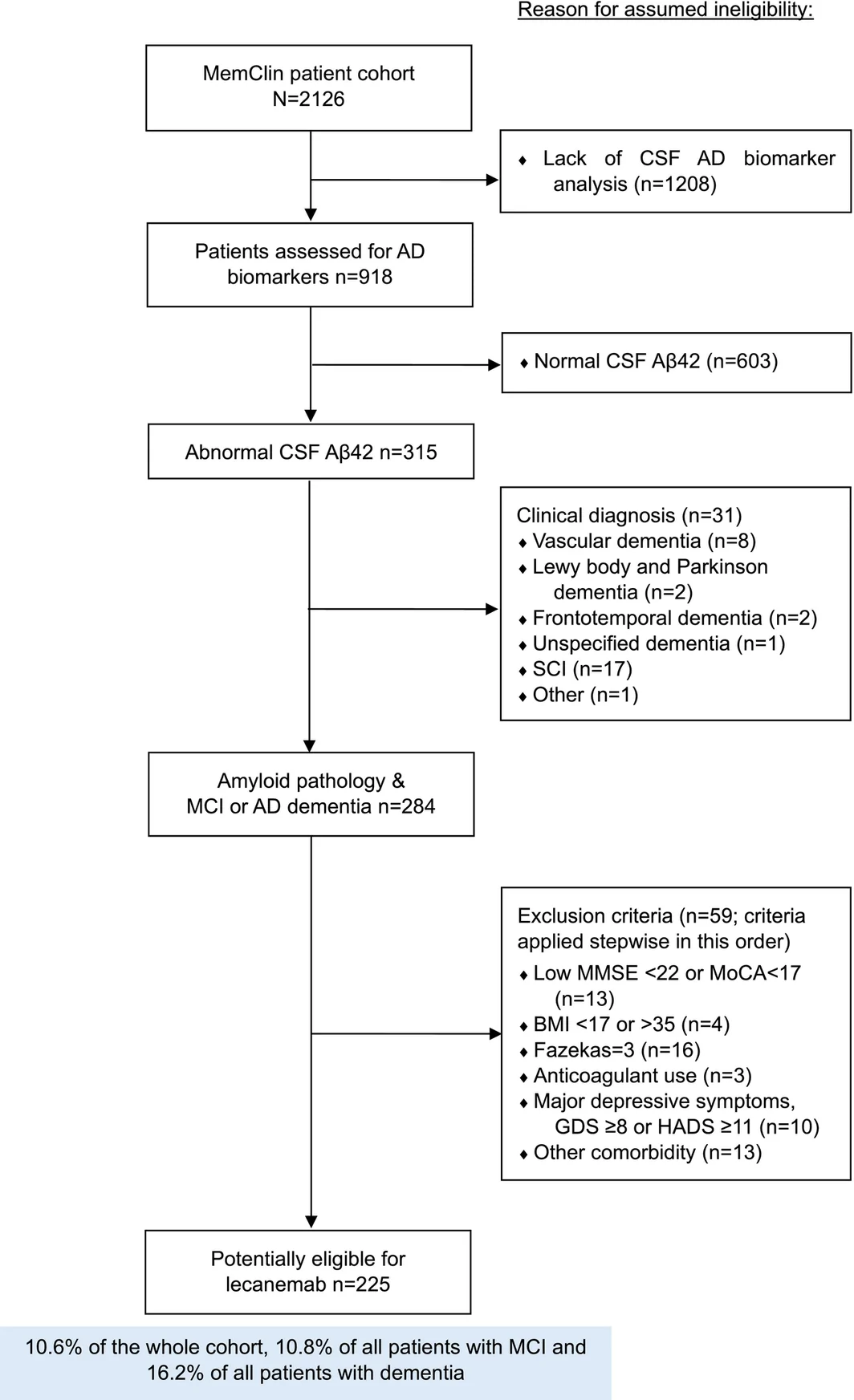
Estimated eligibility for lecanemab. Eligibility was determined according to the appropriate use recommendations (AUR) for lecanemab (Cummings et al., 2023). Abnormal CSF Aβ42 was considered sufficient evidence for AD pathology. Abnormal Aβ42 was defined based on the standard laboratory cut‐offs used in routine clinical practice. The operationalization of the AUR criteria is described in detail in Table S1; not all exclusion criteria could be assessed in our study, including MRI‐related safety criteria (such as cerebral microbleeds, macrohemorrhage, lacunar infarcts, cortical superficial siderosis, and imaging findings suggestive of cerebral amyloid angiopathy). AUR, appropriate use recommendations; AD, Alzheimer's disease; Aβ, amyloid‐β; BMI, body mass index; CSF, cerebrospinal fluid; GDS, Geriatric Depression scale; HADS, Hospital Anxiety and Depression Scale; MCI, mild cognitive impairment; MMSE, Mini‐Mental State Examination; MoCA, Montreal Cognitive Assessment; SCI, subjective cognitive impairment.

**FIGURE 2:**
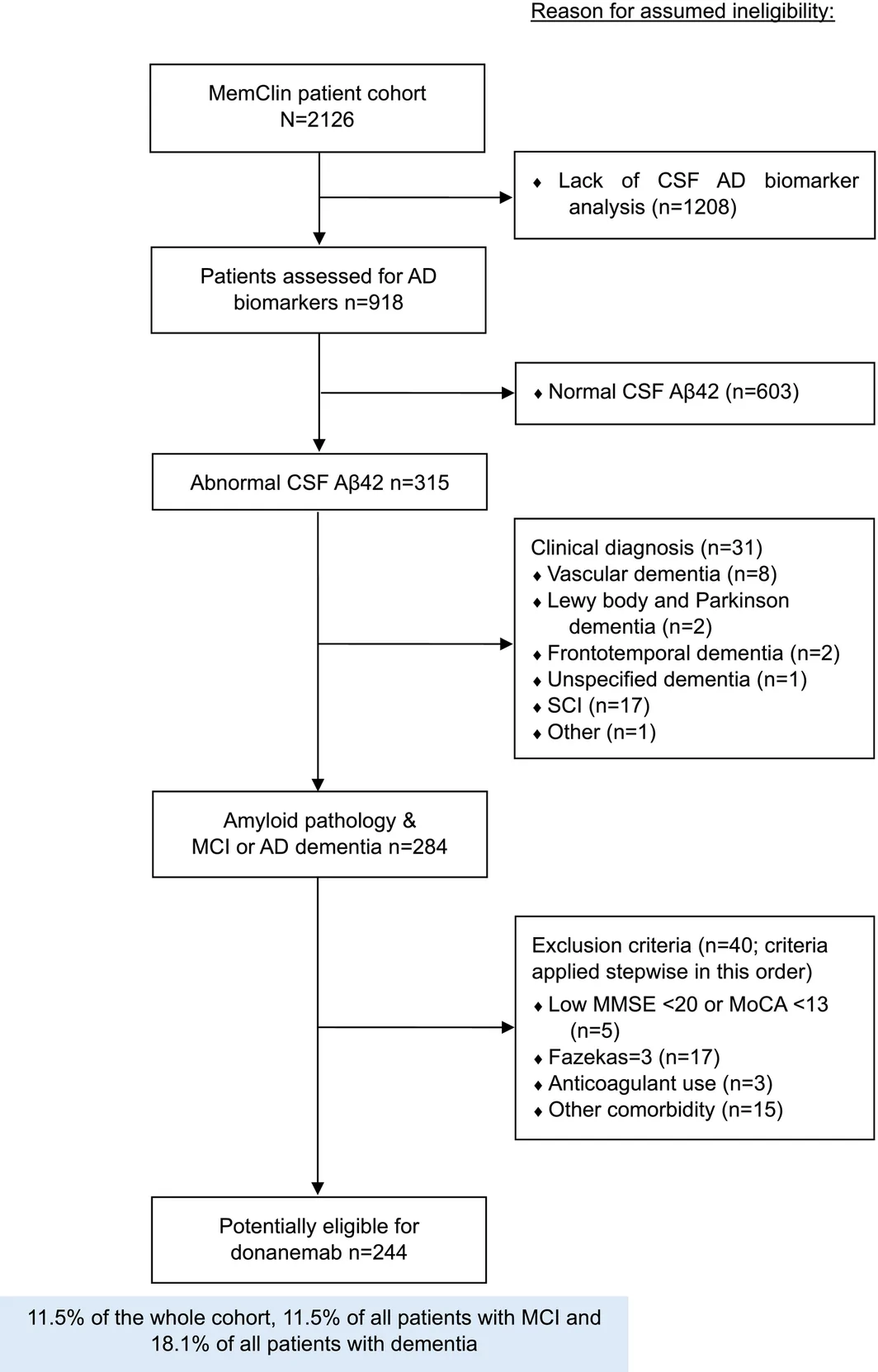
Estimated eligibility for donanemab. Eligibility was determined according to the appropriate use recommendations (AUR) for donanemab (Rabinovici et al., 2025). Abnormal CSF Aβ42 was considered sufficient evidence for AD pathology. Abnormal Aβ42 was defined based on the standard laboratory cut‐offs used in routine clinical practice. The operationalization of the AUR criteria is described in detail in Table S1; not all exclusion criteria could be assessed in our study, including MRI‐related safety criteria (such as cerebral microbleeds, macrohemorrhage, lacunar infarcts, cortical superficial siderosis, and imaging findings suggestive of cerebral amyloid angiopathy). AUR, appropriate use recommendations; AD, Alzheimer's disease; Aβ, amyloid‐β; CSF, cerebrospinal fluid; MCI, mild cognitive impairment; MMSE, Mini‐Mental State Examination; MoCA, Montreal Cognitive Assessment; SCI, subjective cognitive impairment.

Figure 3 shows the percentages of patients meeting each individual exclusion criterion when assessing eligibility for lecanemab and donanemab. The most common single reason for ineligibility was lack of CSF assessment. Approximately half of the patients (49.2%) who were ineligible for both treatments met at least two different exclusion criteria (Figure 3B).

**FIGURE 3:**
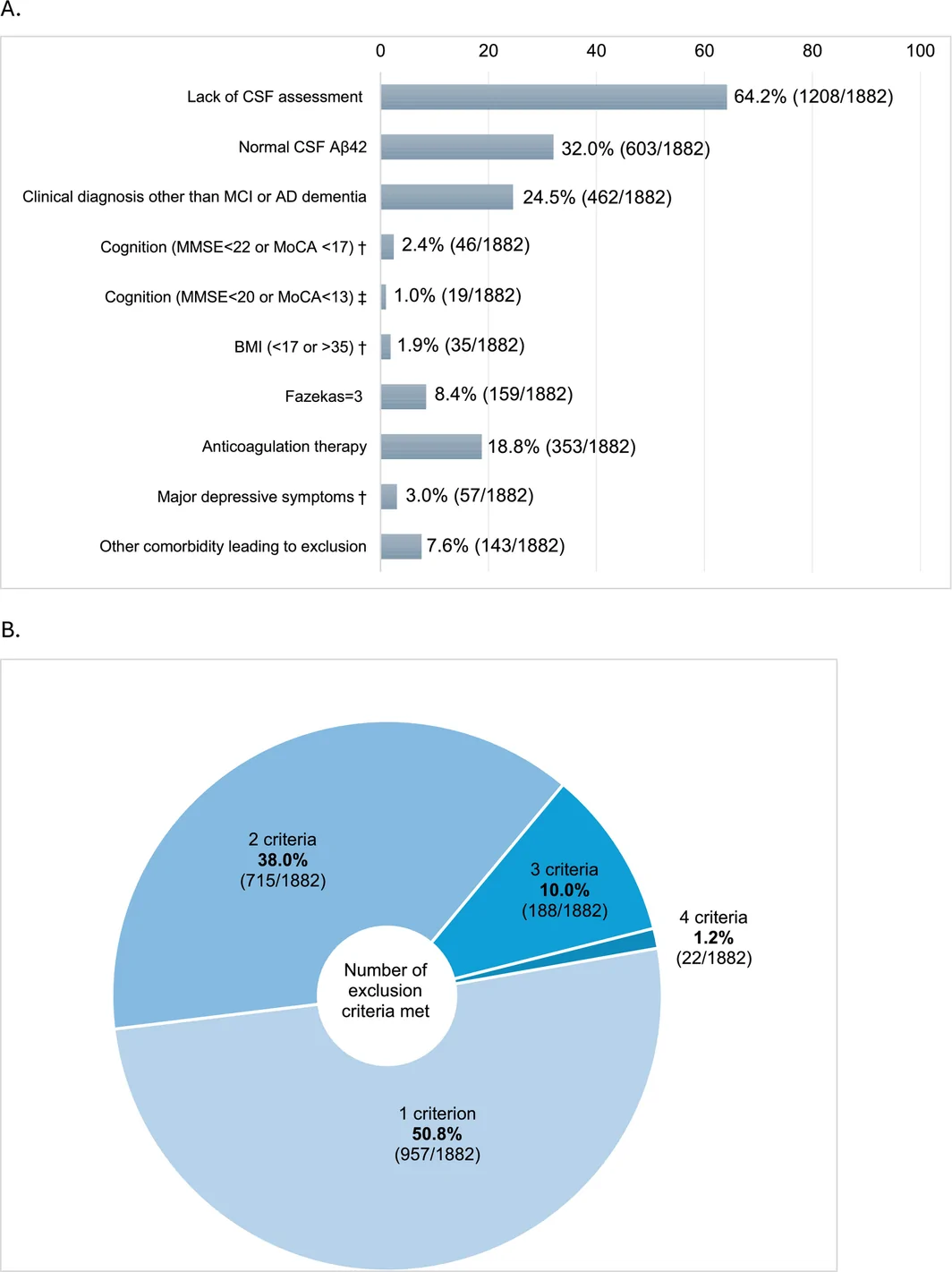
Proportion of patients meeting each exclusion criterion (A) and total number of exclusion criteria met (B), among those who were ineligible for both treatments (*n* = 1882). † Item specific for lecanemab eligibility assessment, ‡ Item specific for donanemab eligibility assessment. AD, Alzheimer's disease; Aβ, amyloid‐β; BMI, body mass index; CSF, cerebrospinal fluid; MCI, mild cognitive impairment; MMSE, Mini‐Mental State Examination; MoCA, Montreal Cognitive Assessment.

Characteristics of patients potentially eligible for either treatment are presented in Table 2. Mean age of eligible patients was 76.3 (SD 5.7) years and 50.0% were women. 61.9% had an MCI‐level diagnosis and 38.1% had dementia. As for biomarker profiles, 31.6% of treatment‐eligible patients were A + T –  N−, 26.3% A + T – N +, 25.4% A + T +  N−, and 16.7% A + T + N +, meaning that the majority, 57.9%, had a normal biomarker of tau pathology (T−; normal CSF p‐tau_181_). Other characteristics are presented in Table 2, together with a comparison to patients who were ineligible for both treatments.

**TABLE 2:** Characteristics of treatment‐eligible patients.

Characteristic	Eligible for either treatment, *n* = 244	Ineligible for both treatments, *n* = 1882	*p*
*Demographics*			
Age, years	76.3 (5.7)	77.2 (6.7)	**0.04**
Women	122 (50.0%)	949 (50.4%)	0.90
Education, years	12.1 (3.5)	12.8 (3.7)	**0.01**
*Clinical diagnosis*			**< 0.001**
SCI	0 (0%)	291 (15.5%)	
MCI	151 (61.9%)	1162 (61.7%)	
Dementia	93 (32.1%)	421 (22.4%)	
*Cognition*			
MMSE, total score	26.5 (2.3)	27.1 (2.4)	**< 0.001**
RAVLT learning	28.1 (8.7)	32.0 (11.2)	**< 0.001**
RAVLT delayed recall (verbal episodic memory)	3.3 (3.3)	5.0 (3.8)	**< 0.001**
RCF copying	28.8 (5.6)	28.9 (5.7)	0.90
RCF immediate recall (non‐verbal episodic memory)	7.2 (5.9)	9.7 (6.8)	**< 0.001**
WAIS‐IV Digit span (working memory)	21.0 (4.8)	21.3 (4.7)	0.34
Verbal fluency, FAS (executive functioning)	34.7 (13.2)	34.4 (14.2)	0.81
WAIS‐IV Block design (visuospatial functions)	27.4 (8.9)	27.7 (9.8)	0.75
WAIS‐IV Information (semantic memory)	12.3 (4.6)	13.8 (4.9)	**< 0.001**
*Clinical characteristics, comorbidity and medical history*			
BMI, kg/m^2^	24.8 (3.8)	25.4 (4.0)	**0.02**
History of stroke/TIA	13 (5.3%)	337 (17.9%)	**< 0.001**
Atrial fibrillation	2 (0.8%)	321 (17.1%)	**< 0.001**
Hypertension diagnosis	119 (48.8%)	955 (50.7%)	0.56
Antihypertensive use	92 (37.7%)	778 (41.3%)	0.28
Hyperlipidemia diagnosis	42 (17.2%)	299 (15.9%)	0.60
Diabetes diagnosis	26 (10.7%)	284 (15.1%)	0.07
Depression diagnosis	24 (9.8%)	193 (10.3%)	0.84
Anticoagulant use	0 (0%)	353 (18.8%)	**< 0.001**
*ATN biomarker classification*			
Biomarker profiles			**< 0.001**
A – T – N −	0 (0%)	227/565 (40.2%)	
A − T – N +	0 (0%)	118/565 (20.9%)	
A − T + N −	0 (0%)	95/565 (16.8%)	
A − T + N +	0 (0%)	60/565 (10.6%)	
A + T – N −	66/209 (31.6%)	22/565 (3.9%)	
A + T − N +	55/209 (26.3%)	22/565 (3.9%)	
A + T + N −	53/209 (25.4%)	14/565 (2.5%)	
A + T + N +	35/209 (16.7%)	7/565 (1.2%)	

*Note:* Data are shown as mean (SD), median [IQR], and *n* (%). ATN classification was based on CSF Aβ42 (A), CSF *p*‐tau_181_ (T), and medial temporal lobe atrophy, MTA (N) (full data for the classification available for *n* = 774). Abnormal CSF biomarkers were defined based on the standard method‐specific cut‐offs used in routine clinical practice. They were: Aβ42 ≤ 550 ng/L (ELISA), ≤ 599 ng/L (ECLIA), and ≤ 620 ng/L (CLEIA); and *p*‐tau ≥ 80 ng/L (ELISA), ≥ 56.5 ng/L (ECLIA), and ≥ 61 ng/L (CLEIA). Abnormal MTA: ≥ 1.5 for patients < 75 years, ≥ 2 for patients aged 75–84 years, and ≥ 2.5 for patients 85+ years. *p*‐values marked in bold are statistically significant < 0.05.

Abbreviations: Aβ, amyloid‐β; ATN, amyloid‐tau‐neurodegeneration (biomarker classification framework); BMI, body mass index; CSF, cerebrospinal fluid; FAS, verbal fluency test with letters F,A,S; MCI, mild cognitive impairment; MMSE, mini‐mental state examination; MTA, medial temporal lobe atrophy; RAVLT, Rey auditory verbal learning test; RCF, Rey complex figure test; TIA, transient ischemic attack; WAIS‐IV, Wechsler adult intelligence scale, fourth edition.

#### 3.2.2 Sensitivity Analyses

Eligibility rates in the scenario where lack of MRI was assumed to equal ineligibility are shown in Figures S6 and S7. In this scenario, 49 patients (2.3% of the whole cohort) met the eligibility criteria for lecanemab, and 58 patients (2.7%) met the eligibility criteria for donanemab. Altogether, 58 patients (2.7%) met the criteria for either treatment, corresponding to 3.0% of all patients with MCI and 3.7% of all patients with dementia (5.3% of all patients with AD‐type dementia).

Estimated eligibility rates for either treatment after accounting for *APOE* genotype (excluding ε4/4 carriers) are shown in Table 3. As *APOE* data were not available in MemClin, we leveraged available data from three other cohorts (two memory clinics [17, 28] and one Swedish population‐based study [29]). We then applied the published estimates of the proportion of otherwise treatment‐eligible patients with ε4/4 genotype to MemClin. In the most permissive scenario (assuming ε4/4 prevalence of 13% [29]), the estimated eligibility was 10.0%. In the most restrictive scenario (assuming ε4/4 prevalence of 46% [17]), the estimated eligibility was 6.2%.

**TABLE 3:** Estimated treatment eligibility when considering *APOE* genotype and excluding ε4/4 carriers (sensitivity analysis).

Assumed % of *APOE* ε4/4 carriers among otherwise eligible patients	*n* (%) of patients who remained eligible for treatment		
	Lecanemab	Donanemab	Either treatment
13% (Dittrich, 2024 [29])	196 (9.2%)	212 (10.0%)	212 (10.0%)
26% (Vigneswaran, 2025 [28])	166 (7.8%)	181 (8.5%)	181 (8.5%)
46% (Rosenberg, 2026 [17])	121 (5.7%)	132 (6.2%)	132 (6.2%)

*Note:* *APOE* data were not available in MemClin. We used therefore published estimates of the proportion of ε4/4 carriers among otherwise treatment‐eligible patients to approximate how many patients in MemClin could remain eligible after excluding ε4/4 carriers (current requirement for treatment eligibility in Europe). The n shown reflects the number of patients who could remain eligible for lecanemab, donanemab, or either of the two treatments, if we assumed that a specific percentage (first column) of otherwise treatment‐eligible patients (lecanemab *n* = 225, donanemab *n* = 244, either treatment *n* = 244) were *APOE* ε4/4 carriers. The % reflects the eligibility rate calculated using these numbers (in the whole MemClin cohort, *n* = 2126 as the denominator).

## 4 Discussion

In this study, we assessed real‐world patient eligibility for anti‐amyloid therapy in a large, naturalistic memory clinic cohort covering seven outpatient memory clinics in Sweden (MemClin). Overall, 11.5% of the cohort met the current appropriate use recommendations (AUR) for either lecanemab or donanemab, corresponding to 11.5% of all patients with MCI and 18.1% of all patients with dementia. This figure should be interpreted as an upper‐bound estimate, as it does not account for all safety exclusions, including MRI‐related contraindications. Eligibility was markedly reduced in a scenario analysis where patients without available MRI data were assumed ineligible, reflecting requirements for imaging‐based safety assessment. Eligibility decreased also when we accounted for *APOE* genotype and excluded ε4/4 carriers by applying estimates of ε4/4 prevalence among otherwise eligible patients from external cohorts to MemClin (resulting in ~6%–10% eligibility, depending on the assumed ε4/4 prevalence). Altogether, our findings suggest that even the upper‐bound estimates of treatment‐eligible patients may currently be relatively low in real‐world memory clinic settings.

Our findings largely align with previous studies, which have mostly reported limited real‐world eligibility for anti‐amyloid therapy [16, 17, 28, 29, 30, 31, 32, 33, 34, 35, 36, 37, 38]. The estimated eligibility rates, however, vary widely depending on the study setting, antibody of interest, and the choice of eligibility criteria and how strictly they are applied (e.g., availability of biomarker testing and MRI data). Eligibility rates tend to be lower in population‐based cohorts than in memory clinic cohorts [29, 31], for lecanemab than for donanemab [35, 36, 37], when clinical trial criteria instead of the more permissive AURs are applied [35, 38], and when biomarker confirmation of amyloid pathology is required for eligibility [33]. Estimates are also strongly influenced by the study population and the denominators used to calculate eligibility rates (e.g., all memory clinic patients vs. clinically selected sample enriched for potential treatment candidates). In unselected memory clinic populations, where many patients have non‐AD cognitive disorders or disease stages outside the treatment indication, reported eligibility rates are generally lower, typically ranging from 2% to 14% [16, 17, 28, 32, 34, 37]. For example, in our earlier work in a single memory clinic, we found 7% eligibility for aducanumab [16], and 6% eligibility for lecanemab and donanemab (3% after excluding *APOE* ε4/4 carriers) [17] among all new patients examined at the clinic. Similar estimates have been reported for example, in Dutch [28], Irish [32], and Korean memory clinic cohorts [37]. In contrast, as observed also in our study, eligibility rates tend to be higher, typically in the range of 15%–50%, when the analysis is restricted to individuals with MCI or AD dementia and/or biomarker‐confirmed Aβ pathology [28, 34, 37, 38, 39]. Local differences in diagnostic and referral pathways and access to biomarker testing further contribute to variation between studies.

In terms of patient characteristics, a key finding of this study was that most treatment‐eligible patients, approximately 58%, had a normal tau biomarker that is, normal CSF *p*‐tau_181_ levels. The biological interpretation of normal tau in our context remains uncertain. While it could suggest that, in real‐world memory clinic settings, anti‐amyloid therapy may predominantly target patients at relatively early disease stages, a large proportion of treatment‐eligible patients in MemClin had a dementia‐stage disease. This indicates that normal CSF *p*‐tau_181_ should not necessarily be interpreted as evidence of early disease. Some individuals may demonstrate biomarker discordance, and p‐tau trajectories may not be strictly linear throughout the disease course [40]. Nevertheless, our findings demonstrate that many treatment‐eligible patients can be tau‐negative by CSF criteria despite confirmed Aβ pathology. Real‐world treatment candidates may thus differ from the participants enrolled in the pivotal clinical trials, such as TRAILBLAZER‐ALZ 2 trial of donanemab which selected a population using tau PET criteria and enriched for tau pathology [2]. While the clinical implications of this observation are unclear, emerging evidence suggests that treatment efficacy and safety may be more favorable at earlier disease stages and in individuals with lower tau burden, highlighting the importance of further characterizing the biomarker profiles of real‐world treatment candidates [2, 41, 42].

While reliable estimates of treatment eligibility are needed to inform healthcare service planning, it is challenging to accurately project the number of patient candidates. Retrospective real‐world eligibility studies, including ours, may overestimate eligibility, as relevant data, such as detailed information on comorbidities and neuroimaging, are usually not easily retrievable. Imaging‐related exclusion criteria are particularly relevant in older populations [31, 33, 37] but can also occur in cohorts with younger memory clinic patients [28]. Detailed assessment of such contraindications requires MRI; however, in many clinical settings, CT remains the first‐line modality for older patients [43], as was also the case in MemClin. CT can be useful to detect neurodegeneration and secondary causes of cognitive impairment when MRI is contraindicated or not available, but in the context of anti‐Aβ treatments, MRI is mandatory for both eligibility assessment and continuous safety monitoring. Expanding MRI capacity will therefore be a critical step in the implementation of anti‐amyloid therapies, and harmonized imaging protocols and sequences sensitive to relevant brain changes will be essential for standardized eligibility and safety assessments.

There are also emerging factors that may substantially influence both eligibility assessments and eligibility rates, particularly over the longer term. Increased awareness of disease‐modifying treatments may lead more individuals with cognitive concerns to seek medical evaluation, thereby increasing the number of diagnoses and patients who can be considered for treatment. The estimated global prevalence of MCI is approximately 15% [44], and it remains an underdiagnosed condition [45]. Blood‐based biomarkers (e.g., plasma p‐tau217) offer a promising alternative to CSF and PET for detecting amyloid pathology and may help streamline eligibility assessments and improve accessibility [46, 47, 48]. Once standardized and validated in different populations, their use may increase the number of patients who can be considered for treatment. Confirmatory CSF/PET testing will nevertheless be needed in some cases, and in the near term, biomarker confirmation in Sweden and in many European countries will likely continue to rely on CSF/PET. The recently updated Finnish Current Care guidelines, for example, state that blood p‐tau217 may be used when CSF sampling is contraindicated and PET is unavailable, but CSF/PET is required if anti‐amyloid therapy is being considered [49].

Our study has several key strengths. We analyzed eligibility for both lecanemab and donanemab in a large, representative multicenter cohort of relatively well‐characterized memory clinic patients. To our knowledge, this is the largest real‐world eligibility study conducted in Europe and the only one including a network of memory clinics. The detailed biomarker profiling of treatment‐eligible patients is another strength, as are the sensitivity analyses exploring eligibility under different scenarios and assumptions. Our findings are highly relevant, as memory clinics within MemClin represent a setting well positioned for early introduction of anti‐amyloid and other disease‐modifying therapies, given the existing diagnostic infrastructure and harmonized dementia diagnostic guidelines.

The main limitations of the study concern missing data in MemClin, primarily on CSF biomarkers and detailed neuroimaging and comorbidity data related to eligibility criteria. While this limits the precision of the eligibility estimates, it reflects current real‐world conditions in many memory clinics. Missing information on MRI findings and other exclusion criteria may have led to an overestimation of eligibility as not all individual exclusion criteria could be applied, whereas classifying patients without CSF biomarker assessment as ineligible may underestimate the proportion of eligible patients. Regarding neuroimaging, most patients underwent CT, and susceptibility‐weighted or T2*‐weighted MRI which are important for assessing imaging‐related exclusion criteria were not routinely performed. Only information on severe white matter changes (Fazekas rating) was systematically available, while other relevant measures, such as cerebral microbleeds and cortical superficial siderosis, could not be assessed. Regarding CSF biomarkers, we assumed patients without CSF data to be ineligible in all analyses, to be able to provide informative estimates of near‐term treatable patients. Lumbar puncture could be omitted from routine diagnostic assessments in our cohort for example, due to contraindications, excessive patient burden, or when the suspected diagnosis was not AD, all of which may also indicate treatment ineligibility. Our results indeed showed that patients who did not undergo a lumbar puncture were more likely to use anticoagulants and have comorbidity that could prevent treatment with anti‐amyloid therapy. However, some of these patients may have had underlying amyloid pathology and could potentially have become treatment candidates following further diagnostic workup. Our estimates therefore reflect both biological eligibility for anti‐amyloid therapy and the current healthcare‐system readiness to identify eligible patients.

A further limitation in MemClin is that eligibility could not be estimated specifically among patients with clinically suspected AD at the time of referral, and *APOE* data were not available. Our adjusted eligibility estimates after excluding ε4/4 carriers therefore represent modeled estimates based on data from external cohorts rather than directly observed values. Given their younger patient populations, ε4/4 prevalence may be higher in the two external memory clinic cohorts than in MemClin, and lower in the population‐based cohort. Finally, as experience with anti‐amyloid therapies accumulates and treatment recommendations evolve, eligibility estimates based on the current criteria and AURs may not fully reflect future clinical practice. Importantly, eligibility decisions should be based on an individualized and comprehensive assessment of a patient's status rather than any single criterion alone.

## 5 Conclusion

In this large cohort of patients evaluated for cognitive symptoms at seven Swedish memory clinics, approximately up to 12% were potentially eligible for anti‐amyloid therapy. Even this upper‐bound estimate suggests that the eligible patient group may be substantially smaller than the overall patient population currently referred to specialist memory clinics for cognitive assessment. Our findings provide timely evidence to inform the debate on public healthcare adoption of anti‐amyloid therapy, particularly following the recent decisions against their use in some countries, such as Sweden.

## Author Contributions

**Anna Rosenberg:** conceptualization, methodology, formal analysis, writing – review and editing, writing – original draft, visualization, investigation, data curation, validation, project administration, software. **Anna Rennie:** investigation, data curation, methodology, project administration, writing – review and editing. **Urban Ekman:** investigation, methodology, data curation, project administration, writing – review and editing. **Makrina Daniilidou:** writing – review and editing, project administration, methodology. **Anette Hall:** writing – review and editing, software, formal analysis, methodology. **Anna Matton:** funding acquisition, writing – review and editing, project administration, resources, methodology. **Ari Solomon:** conceptualization, methodology, supervision, writing – review and editing. **Lars‐Olof Wahlund:** investigation, data curation, project administration, writing – review and editing. **Miia Kivipelto:** conceptualization, writing – review and editing, project administration, supervision, funding acquisition, resources. **Eric Westman:** conceptualization, investigation, methodology, supervision, project administration, resources, data curation, funding acquisition, writing – review and editing.

## Funding

This work was supported by the Innovative Health Initiative Joint Undertaking (JU) under grant agreement No 101112145. The JU receives support from the European Union's Horizon Europe research and innovation programme and COCIR, EFPIA, EuropaBio, MedTech Europe, Vaccines Europe, BioArctic and Combinostics Oy. Disclaimer: Funded by the European Union, the private members, and those contributing partners of the IHI JU. Views and opinions expressed are, however, those of the author(s) only and do not necessarily reflect those of the aforementioned parties. Neither of the aforementioned parties can be held responsible for them. This project was also supported by the FORTE research center grant 2025‐02071—“KI Transdisciplinary Research Center for Personalized Dementia Prevention & Care (TraCeDem)”. MK received funding from the Innovative Health Initiative Joint Undertaking (IHI JU)‐PROMINENT under grant agreement No. 101112145; EU Innovative Health Initiative Joint Undertaking (IHI JU) AD‐RIDDLE under grant agreement No. 101132933; Alzheimer's Drug Discovery Foundation (USA); Region Stockholm research grant (ALF, Sweden); Center for Innovative Medicine (CIMED) at Karolinska Institute (Sweden); Stiftelsen Stockholms sjukhem (Sweden); Swedish research council for health, working life and welfare (FORTE). AS received funding from the EU Joint Programme—Neurodegenerative Disease Research (JPND) Multi‐MeMo grant (Academy of Finland No. 357810); Innovative Health Initiative Joint Undertaking (IHI JU) ‐PROMINENT under grant agreement No. 101112145; EU Innovative Health Initiative Joint Undertaking (IHI JU) AD‐RIDDLE, under grant agreement No. 101132933; Alzheimerfonden (Sweden); Juho Vainio Foundation (Finland); Finnish Cultural Foundation (Finland); Yrjö Jahnsson Foundation (Finland). EW received funding from the Swedish Research Council (VR) No. 2025‐02405, the Center for Innovative Medicine (CIMED) No. FoUI‐104172, the regional agreement on medical training and clinical research (ALF) between Stockholm County Council and Karolinska Institutet No. FoUI‐952838, FoUI‐954893, The Swedish Brain Foundation (Hjärnfonden) No. FO2026‐0228, The Swedish Alzheimer's Foundation (Alzheimerfonden) No. AF‐1031812, The Swedish Parkinson's foundation (Parkinsonfonden) No. 1647/25, EU Innovative Health Initiative Joint Undertaking (IHI JU) AD‐RIDDLE and ACCESS AD, King Gustaf V:s and Queen Victorias Foundation, Olle Engkvists Foundation (Olle Engkvists Stiftelse), Sweden's Innovation Agency (VINNOVA) 2025‐03749, and The Swedish Dementia Foundation (Demensfonden). The funders had no role in the design and conduct of the study; in the collection, analysis, and interpretation of data; in the preparation of the manuscript; or in the review or approval of the manuscript.

## Disclosure

*Declaration of the Use of Generative AI and AI‐Assisted Technologies*: No generative AI or AI‐assisted technologies were used in the writing process or in the figures of this manuscript. ChatGPT was used to help draft the graphical abstract image.

## Ethics Statement

All participants provided an informed consent, and the MemClin study has an ethical approval (Regional Ethical Review Board in Sweden; Dnr: 2016/29–31/1).

## Conflicts of Interest

M.K. has served on scientific advisory boards at Eisai, BioArctic, Eli Lilly, Nestlé, and Combinostics. All the other authors declare no conflicts of interest.

## Supporting information


**Figure S1:** ATN biomarker profiles of patients in the MemClin cohort.
**Figure S2:** Estimated eligibility for lecanemab among patients with MCI.
**Figure S3:** Estimated eligibility for lecanemab among patients with dementia.
**Figure S4:** Estimated eligibility for donanemab among patients with MCI.
**Figure S5:** Estimated eligibility for donanemab among patients with dementia.
**Figure S6:** Estimated eligibility for lecanemab (sensitivity analysis, assuming all patients without MRI to be ineligible).
**Figure S7:** Estimated eligibility for donanemab (sensitivity analysis, assuming all patients without MRI to be ineligible).
**Table S1:** The Appropriate Use Recommendations (AUR) patient eligibility criteria for lecanemab^1^ and their operationalization in our study.
**Table S2:** The Appropriate Use Recommendations (AUR) patient eligibility criteria for donanemab^1^ and their operationalization in our study.
**Table S3:** CSF and imaging biomarkers of the MemClin patient cohort.
**Table S4:** Comparison of patients with and without CSF biomarker data.

## Data Availability

The MemClin data, while pseudonymized, is considered personal data and is therefore not publicly available. The data may only be available upon reasonable request if the legal and ethical requirements can be met.
