# Supplementary material for: Real‐World Patient Eligibility for Alzheimer's Disease Anti‐Amyloid Therapy in Seven Swedish Memory Clinics
Source: Eur J Neurol. 2026 Oct 20;33(10):e70786. doi: 10.1111/ene.70786 (PMC13681011; doi:10.1111/ene.70786)
Supplement: Supplementary file 1 [file ENE-33-e70786-s001.docx]

**SUPPLEMENTARY MATERIAL**

**Supplementary Table 1.** The Appropriate Use Recommendations (AUR) patient eligibility criteria for lecanemab^1^ and their operationalization in our study

| **Eligibility criteria** | **Operationalization in our dataset** |
| --- | --- |
| **Inclusion criteria** | |
| Presence of amyloid pathology:  Positive amyloid PET or CSF indicative of AD | Abnormal CSF Aβ42, based on the cut-offs provided by the laboratory/manufacturer and routinely used in clinical practice* |
| Diagnosis: clinical diagnosis of MCI or  mild AD dementia | Clinical diagnosis of MCI (ICD-10 F06.7; no evidence of a non-AD neurological disorder) or mild AD dementia (ICD-10 F00, G30) |
| Age: Physician judgement used outside the 50–90-y. range | No exclusion based on age |
| Global cognition: MMSE 22–30 or other screening instrument with a score compatible with early AD | MMSE 22–30, or if MMSE missing, MoCA 17–30. Patients with missing data on both cognitive screening assessments were not excluded. |
| Body mass index: Physician judgement used at extreme values (clinical trial criteria: >17 kg/m^2^ and <35 kg/m^2^) | Body mass index (BMI) >17 and <35 kg/m^2^; those with missing BMI data were not excluded. |
| Patients may be on cognitive enhancing agents (donepezil, rivastigmine, galanta­mine, or memantine) for AD, but not on aducanumab | Participants with or without acetylcholinesterase inhibitors/memantine were included |
| Patients may be on standard of care for other medical illnesses (see below for specifics regarding anticoagulation) | Participants with or without standard care for other medical conditions were included (see below regarding anticoagulation) |
| Have a care partner or family member(s) who can ensure that the patient has the support needed to be treated with lecanemab | NA |
| Patients, care partners, and appropriate family members should understand the requirements for lecanemab therapy and the potential benefit and potential harm of treatment | NA |
| *APOE* genotyping of all treatment candidates is recommended before initiating lecanemab therapy | NA*. APOE* genotyping is currently not part of the standard workflow of dementia diagnostics (performed only in some clinics for selected patients).  As a sensitivity analysis, we used published estimates of the proportion of ε4/4 carriers among otherwise treatment-eligible patients in three external cohorts^2-4^ to approximate eligibility rates in MemClin after excluding ε4/4 carriers (European regulatory requirement) |
| **Exclusion criteria** | |
| Any medical, neurologic, or psychiatric condition that may be contributing to the cognitive impairment or any non-AD MCI or dementia | Patients with diagnoses other than MCI or AD dementia were excluded. |
| Recent history (within 12 months) of stroke or transient ischemic attacks or any history of seizures  Mental illness (e.g., psychosis), or major depression that interferes with comprehension of the requirements, potential benefit, and potential harms of treatment and are considered by the physician to render the patient unable to comply with management requirements.  Any history of immunologic disease (e.g., lupus erythematosus, rheumatoid arthritis, Crohn’s disease) or systemic treatment with immunosuppressants, immunoglobulins, or monoclonal antibodies or their derivatives  Patients with a bleeding disorder that is not under adequate control.  Unstable medical conditions that may affect or be affected by lecanemab therapy. | Detailed medical history and status of current medical conditions were not available to fully assess all exclusion criteria related to comorbidity, e.g., immunologic comorbidities or prior seizures, or e.g., the management status of common conditions like hypertension. Major medical conditions (e.g., cardiovascular, psychiatric, depression, cancer) are nevertheless usually stabilized before referral to the memory clinics, or before the referral is accepted. Patient medical history/comorbid conditions are also considered in the diagnostics. Patients with a history of stroke or TIA were thus not excluded as data on the time of the event was not available (likely >12 months prior to visit due to the referral criteria). Patients with psychiatric conditions of severe or chronic nature, and e.g., brain tumor were excluded. Depression: patients with a GDS score ≥8 or HADS score ≥11 were excluded (indicating moderately severe/severe depression; those with missing assessment were not excluded). |
| Patients on anticoagulants should not receive lecanemab; tPA should not be administered to individuals on lecanemab.  Use of concomitant aspirin and other antiplatelet therapy permitted (treatment guidelines in Europe). | Patients with anticoagulant treatment other than aspirin/antiplatelet therapy were excluded. |
| Inability to undergo MRI due to claustrophobia, pacemaker, defibrillator, or metal implants  MRI evidence of a non-AD dementia  More than 4 microhemorrhages (defined as 10 mm or less at the greatest diameter); a single macrohemorrhage >10 mm at greatest diameter; an area of superficial siderosis; evidence of vasogenic edema; more than 2 lacunar infarcts or stroke involving a major vascular territory; severe subcortical hyperintensities consistent with a Fazekas score of 3; evidence of amyloid beta-related angiitis; cerebral amyloid angiopathy-related inflammation; or other major intracranial pathology that may cause cognitive impairment. | Most patients in this cohort underwent CT instead of MRI (CT is part of the basic dementia examination in primary care). Patients without MRI were not excluded in the main analysis, because lack of MRI in this dataset did not necessarily imply medical contraindications. MRI might have been performed to complement the examination depending on the clinic and patient, but this was not always the case.  As MRI is mandatory when screening for eligibility for anti-amyloid therapy, we performed a sensitivity analysis by excluding all patients without MRI.  Patients diagnosed with conditions other than MCI/AD dementia (considering all available data incl. imaging) were excluded.  Patients with Fazekas =3 were excluded (based on CT or MRI). Data on the other exclusion criteria, including number and distribution of cerebral microbleeds; macrohemorrhages; presence of cortical superficial siderosis; number and location of lacunar infarcts; or imaging patterns suggestive of cerebral amyloid angiopathy, were not available for the eligibility assessment. This is due to the limited quality of imaging data in this real-world memory clinic patient cohort (only CT was performed, or MRI sequences were limited e.g., no routine susceptibility-weighted imaging (SWI) or T2*-weighted imaging). |

Eligibility was assessed according to the lecanemab appropriate use recommendations (AUR)^1^. Abnormal CSF Aβ42 regardless of tau status was considered sufficient evidence for AD pathology, in line with the European treatment guidelines (confirmed Aβ pathology). Inclusion and exclusion criteria are listed on the left, and the right column shows how the criteria were applied in our data to define eligibility. NA indicates that the item was not considered in the analysis (information not available).

* Depending on the clinic, CSF Aβ42 was analyzed with ELISA, ECLIA, or CLEIA assays. Cut-offs for abnormal levels were as follows: ≤550 ng/l (ELISA), ≤599 ng/l (ECLIA), and ≤620 ng/l (CLEIA).

Abbreviations: AD=Alzheimer’s disease; APOE=apolipoprotein E; AUR=Appropriate Use Recommendations; Aβ=β-amyloid; CSF=cerebrospinal fluid; CT=computed tomography; GDS=Geriatric Depression Scale; HADS= Hospital Anxiety and Depression Scale; MCI=mild cognitive impairment; MMSE=Mini-Mental State Examination; MoCA=Montreal Cognitive Assessment; PET=positron emission tomography; TIA=transient ischemic attack.

References:

1. Cummings J, et al. Lecanemab: Appropriate Use Recommendations. J Prev Alzheimers Dis. 2023;10(3):362-377. doi: 10.14283/jpad.2023.30.
2. Rosenberg A, et al. Preparing for the implementation of anti-amyloid therapies in Europe: Assessing real-world eligibility for lecanemab and donanemab in a Swedish memory clinic. J Prev Alzheimers Dis. 2026 Mar;13(3):100476. doi: 10.1016/j.tjpad.2025.100476.
3. Vigneswaran S, et al. "Real-world" eligibility for anti-amyloid treatment in a tertiary memory clinic setting. Alzheimers Dement. 2025 Jun;21(6):e70375. doi: 10.1002/alz.70375.
4. Dittrich A, et al. Proportion of Community-Dwelling Individuals Older Than 70 Years Eligible for Lecanemab Initiation: The Gothenburg H70 Birth Cohort Study. Neurology. 2024 May 14;102(9):e209402. doi: 10.1212/WNL.0000000000209402.

**Supplementary Table 2.** The Appropriate Use Recommendations (AUR) patient eligibility criteria for donanemab^1^ and their operationalization in our study

| **Eligibility criteria** | **Operationalization in our dataset** |
| --- | --- |
| **Inclusion criteria** | |
| Presence of amyloid pathology:  Positive amyloid PET or CSF indicative of AD pathology | Abnormal CSF Aβ42, based on the cut-offs provided by the laboratory/manufacturer and routinely used in clinical practice* |
| Diagnosis: clinical diagnosis of MCI or dementia with mild functional impairment, with AD as the suspected etiology based on cognitive-behavioral syndrome | Clinical diagnosis of MCI (ICD-10 F06.7; no evidence of a non-AD neurological disorder) or mild AD dementia (ICD-10 F00, G30) |
| Age: Physician judgement used for patients outside the 60–85-year age range | No exclusion based on age |
| Global cognition: MMSE 20–30, MoCA 13–30, or other cognitive screening instrument with a score compatible with early AD | MMSE 20–30, or if MMSE missing, MoCA 13–30. Patients with missing data on both cognitive screening assessments were not excluded. |
| Patients may be on cognitive enhancing agents for AD (donepezil, rivastigmine, galantamine, memantine), but not on aducanumab or lecanemab | Participants with or without acetylcholinesterase inhibitors/memantine were included |
| Patients may be on standard of care for other medical illnesses (see below for specifics regarding anticoagulation) | Participants with or without standard care for other medical conditions were included (see below regarding anticoagulation) |
| Patients should understand that the effect of donanemab on the ability to have children or its effect on the unborn fetus are unknown | NA |
| Have a care partner or family member(s) who can ensure that the patient has the support needed to be treated with donanemab | NA |
| In the opinion of the clinician, have adequate literacy, vision, and hearing for cognitive testing | MemClin cohort consists of patients who underwent a neuropsychological assessment; no impact on eligibility analysis. |
| Treated patients are reliable and are willing to follow study procedures | NA |
| Patients, care partners, and appropriate family members understand the requirements for donanemab therapy and the potential benefits and harms of treatment. Approval by surrogate decision maker with patient assent may be appropriate if patient lacks capacity to make medical decisions independently | NA |
| *APOE* genotyping is performed prior to initiating treatment to assess an individual's risk of ARIA | NA*. APOE* genotyping is currently not part of the standard workflow of dementia diagnostics (performed only in some clinics for selected patients)  As a sensitivity analysis, we used published estimates of the proportion of ε4/4 carriers among otherwise treatment-eligible patients in three external cohorts^2-4^ to approximate eligibility rates in MemClin after excluding ε4/4 carriers (European regulatory requirement) |
| **Exclusion criteria** | |
| Non-AD neurologic condition that may be significantly contributing to cognitive or behavioral impairment | Patients with diagnoses other than MCI or AD dementia were excluded. |
| Autosomal dominant AD, if the mutation is associated with high prevalence and burden of cerebral amyloid angiopathy (CAA) | NA. Genetic testing for familial AD is not routinely conducted, no information about potential autosomal dominant AD cases. |
| AD due to Down syndrome | No patients with Down syndrome in this cohort. No impact on eligibility analysis. |
| Recent history (within 12 months) of stroke or transient ischemic attacks or any history of seizures  Psychiatric disorder, suicidal ideation, or history of alcohol or substance use that interferes with comprehension of the requirements, potential benefit, and potential harms of treatment and are considered by the clinician to render the patient unable to comply with treatment requirements  Medical condition that may significantly contribute to cognitive impairment or interfere with the patients’ ability to participate in treatment or the clinician's ability to assess the patient  Physical condition that impairs the ability of the patient to have intravenous infusions  Patients with known allergies to donanemab or related compounds are excluded  History of immunologic disease (e.g., systemic lupus erythematosus, rheumatoid arthritis, Crohn's disease) or current systemic treatment with immunosuppressants, immunoglobulins, or monoclonal antibodies or their derivatives  Active cancer that interferes with the ability to comply with donanemab treatment  Patients with a bleeding disorder that is not under adequate control.  Findings on physical or neurological examination, vital signs, or laboratory tests that may significantly contribute to cognitive impairment or interfere with the patients’ ability to participate in treatment or the clinician's ability to assess the patient | Detailed medical history and status of current medical conditions were not available to fully assess all exclusion criteria related to comorbidity, e.g., immunologic comorbidities, or prior seizures, or e.g., the management status of common conditions like hypertension. Major medical conditions (e.g., cardiovascular, psychiatric, depression, cancer) are nevertheless usually stabilized before referral to the memory clinics, or before the referral is accepted. Patient medical history/comorbid conditions are also considered in the diagnostics. Patients with a history of stroke or TIA were thus not excluded as data on the time of the event was not available (likely >12 months prior to visit due to the referral criteria). Patients with psychiatric conditions of severe or chronic nature, and e.g., brain tumor were excluded. |
| If previously treated with an Aβ-targeting therapy, must allow washout period of >5 half-lives; must demonstrate current biomarker evidence of Aβ plaques, and must not meet other clinical or MRI exclusion criteria | No history of anti-Aβ treatment in this cohort. No impact on eligibility analysis. |
| Patients on anticoagulants (coumadin, dabigatran, rivaroxaban, apixaban, betrixaban, or heparin) should not receive donanemab; thrombolytics should not be administered to individuals on donanemab | Patients with anticoagulant treatment other than aspirin/antiplatelet therapy were excluded. |
| Sensitivity or contraindication to amyloid imaging ligands if amyloid PET is required for confirmation of AD | CSF used to determine amyloid pathology in this cohort. No impact on eligibility analysis. |
| Contraindications for MRI, incl. claustrophobia or the presence of contraindicated metal (ferromagnetic) implants/cardiac pacemaker  Abnormality suggesting a non-AD cause for progressive cognitive impairment or a clinically significant finding that may impact safety  Amyloid-related imaging abnormalities of edema/effusion, more than 4 cerebral micro-hemorrhages, any area of superficial siderosis; any intracerebral hemorrhage greater than 1 cm or severe white matter disease; CAA-related inflammation; evidence of territorial infarcts > 1 cm; more than 2 lacunar infarcts, cerebral contusion, encephalomalacia, brain aneurysms or other vascular malformations, central nervous system infection, and brain tumors, except for small meningiomas or arachnoid cysts. | Most patients in this cohort underwent CT instead of MRI (CT is part of the basic dementia examination in primary care). Patients without MRI were not excluded in the main analysis, because lack of MRI in this dataset did not necessarily imply medical contraindications. MRI might have been performed to complement the examination depending on the clinic and patient, but this was not always the case.  As MRI is mandatory when screening for eligibility for anti-amyloid therapy, we performed a sensitivity analysis by excluding all patients without MRI.  Patients diagnosed with conditions other than MCI/AD dementia (considering all available data incl. imaging) were excluded.  Patients with Fazekas =3 were excluded (based on CT or MRI).  Data on the other exclusion criteria, including e.g., number and distribution of cerebral microbleeds; macrohemorrhages; presence of cortical superficial siderosis; number and location of lacunar infarcts; or imaging patterns suggestive of cerebral amyloid angiopathy, were not available for the eligibility assessment. This is due to the limited quality of imaging data in this real-world memory clinic patient cohort (only CT was performed, or MRI sequences were limited e.g., no routine susceptibility-weighted imaging (SWI) or T2*-weighted imaging). |

Eligibility was assessed according to the donanemab appropriate use recommendations (AUR)^1^. Abnormal CSF Aβ42 regardless of tau status was considered sufficient evidence for AD pathology, in line with the European requirements and guidelines by EMA (confirmed Aβ pathology). Inclusion and exclusion criteria are listed on the left, and the right column shows how the criteria were applied in our data to define eligibility. NA indicates that the item was not considered in the analysis (information not available).

* Depending on the clinic, CSF Aβ42 was analyzed with ELISA, ECLIA, or CLEIA assays. Cut-offs for abnormal levels were as follows: ≤550 ng/l (ELISA), ≤599 ng/l (ECLIA), and ≤620 ng/l (CLEIA).

Abbreviations: AD=Alzheimer’s disease; APOE=apolipoprotein E; AUR=Appropriate Use Recommendations; Aβ=β-amyloid; CSF=cerebrospinal fluid; CT=computed tomography; MCI=mild cognitive impairment; MMSE=Mini-Mental State Examination; MoCA=Montreal Cognitive Assessment; PET=positron emission tomography; TIA=transient ischemic attack.

References:

1. Cummings J, et al. Lecanemab: Appropriate Use Recommendations. J Prev Alzheimers Dis. 2023;10(3):362-377. doi: 10.14283/jpad.2023.30.
2. Rosenberg A, et al. Preparing for the implementation of anti-amyloid therapies in Europe: Assessing real-world eligibility for lecanemab and donanemab in a Swedish memory clinic. J Prev Alzheimers Dis. 2026 Mar;13(3):100476. doi: 10.1016/j.tjpad.2025.100476.
3. Vigneswaran S, et al. "Real-world" eligibility for anti-amyloid treatment in a tertiary memory clinic setting. Alzheimers Dement. 2025 Jun;21(6):e70375. doi: 10.1002/alz.70375.
4. Dittrich A, et al. Proportion of Community-Dwelling Individuals Older Than 70 Years Eligible for Lecanemab Initiation: The Gothenburg H70 Birth Cohort Study. Neurology. 2024 May 14;102(9):e209402. doi: 10.1212/WNL.0000000000209402.

**Supplementary Table 3.** CSF and imaging biomarkers of the MemClin patient cohort

|  | **n with data** | **All**  **(N=2126)** | **SCI**  **(n=291)** | **MCI**  **(n=1313)** | **Dementia (n=514)** | **p-value** |
| --- | --- | --- | --- | --- | --- | --- |
| **CSF biomarkers** | | | | | | |
| Aβ42, ng/l  ELISA  ECLIA  CLEIA | 708  149  61 | 708 (287)  822 (439)  829 (379) | 875 (302)  1231 (531)  1217 (443) | 718 (279)  839 (439)  788 (370) | 609 (253)  596 (201)  716 (259) | **<0.001**  **<0.001**  **0.03** |
| Aβ42/40  ELISA  ECLIA  CLEIA | 124  143  58 | 0.6 (0.3)  0.7 (0.3)  0.7 (0.3) | 0.8 (0.2)  0.9 (0.2)  0.9 (0.2) | 0.6 (0.3)  0.7 (0.3)  0.6 (0.3) | 0.6 (0.3)  0.5 (0.1)  0.6 (0.3) | **0.03**  **<0.001**  0.06 |
| p-tau_181_, ng/l  ELISA  ECLIA  CLEIA | 711  152  64 | 67 (42)  75 (47)  76 (41) | 56 (21)  44 (19)  46 (16) | 66 (48)  68 (39)  75 (44) | 73 (35)  111 (59)  93 (35) | **<0.001**  **<0.001**  **0.01** |
| t-tau, ng/l  ELISA  ECLIA  CLEIA | 710  152  64 | 455 (253)  503 (297)  531 (272) | 374 (199)  294 (154)  408 (189) | 437 (232)  473 (260)  515 (287) | 526 (297)  686 (366)  632 (250) | **<0.001**  **<0.001**  0.07 |
| NFL, ng/l | 342 | 1499 (1216) | 1166 (1413) | 1465 (1089) | 1701 (1299) | **<0.001** |
| **Imaging biomarkers** | | | | | |  |
| MTA rating, median [IQR] | 1744 | 1 [1–2] | 1 [0–1] | 1 [1–2] | 2 [1–2.5] | **<0.001** |
| Fazekas rating, median [IQR] | 1737 | 1 [0.5–2] | 1 [0–1.5] | 1 [0.5–2] | 1 [0.5–2] | **<0.001** |
| GCA rating, median [IQR] | 1745 | 1 [1–2] | 1 [0.5–1.5] | 1 [1–2] | 1 [1–2] | **<0.001** |

Data are shown as mean (SD)or median [IQR] . CSF biomarkers (measured with any method) were available for total n=918 (SCI n=113, MCI n=547, dementia n=253; n=5 with other clinical diagnoses). Imaging (CT or MRI) was performed for n=2060 (SCI n=285, MCI n=1268, dementia n=500; n=7 with other clinical diagnoses). P-values marked in bold are statistically significant <0.05, indicating differences in biomarkers among the three diagnostic groups. Cut-offs for CSF biomarker abnormality (provided by the laboratory/manufacturer): for Aβ42 ≤550 ng/L (ELISA), ≤599 ng/L (ECLIA), and ≤620 ng/L (CLEIA); for Aβ42/40 ≤0.89 (ELISA), ≤0.68 (ECLIA), and ≤0.61 (CLEIA); for p-tau_181_ ≥80 ng/L (ELISA), ≥56.5 ng/L (ECLIA), and ≥61 ng/L (CLEIA); for total tau ≥400 ng/l (ELISA), ≥404 ng/l (ECLIA), and ≥360 ng/l (age <50 years) and ≥479 ng/l (age 50+ years) (CLEIA); and for NFL ≥380 ng/l (age <30 years), ≥560 ng/l (age 30-39 years), ≥890 ng/l (age 40-59 years) and ≥1850 ng/l (age 60+ years).

Abbreviations: Aβ=amyloid-β; CSF=cerebrospinal fluid; GCA=global cortical atrophy; IQR=interquartile range; MCI=mild cognitive impairment; MTA=medial temporal lobe atrophy; NFL=neurofilament light; p-tau=phosphorylated tau; SCI=subjective cognitive impairment; t-tau=total tau.

**Supplementary Table 4.** Comparison of patients with and without CSF biomarker data

| **Characteristic** | **n with data** | **No CSF data**  **n=1208** | **CSF data available**  **n=918** | **p-value** |
| --- | --- | --- | --- | --- |
| **Demographics** | | | | |
| Age, years | 2126 | 78.1 (6.7) | 75.7 (6.3) | **<0.001** |
| Women | 2126 | 616 (51.0%) | 455 (49.6%) | 0.51 |
| Education, years | 1805 | 12.8 (3.6) | 12.7 (3.7) | 0.34 |
| Clinical diagnosis  SCI  MCI  Dementia | 2126 | 178 (14.7%)  766 (63.4%)  261 (21.6%) | 113 (12.3%)  547 (59.6%)  253 (27.6%) | **0.01** |
| **Cognition** | | | | |
| MMSE | 1989 | 27.1 (2.3) | 26.9 (2.5) | 0.09 |
| RAVLT learning, total score | 1406 | 31.5 (10.9) | 31.7 (11.3) | 0.74 |
| RAVLT delayed recall  (verbal episodic memory) | 1015 | 4.9 (3.7) | 4.8 (3.9) | 0.40 |
| RCF copying, total score | 1452 | 28.8 (5.9) | 29.0 (5.5) | 0.73 |
| RCF immediate recall  (non-verbal episodic memory) | 1350 | 9.4 (6.7) | 9.4 (6.8) | 0.89 |
| WAIS-IV Digit span  (working memory) | 1441 | 21.3 (4.7) | 21.3 (4.7) | 0.86 |
| Verbal fluency, FAS  (executive functioning) | 1517 | 34.2 (14.0) | 34.7 (14.2) | 0.71 |
| WAIS-IV Block design (visuospatial functions) | 1365 | 27.2 (9.6) | 28.1 (9.8) | 0.11 |
| WAIS-IV Information  (semantic memory) | 1608 | 13.7 (4.9) | 13.5 (4.8) | 0.45 |
| **Clinical characteristics, comorbidity and medical history** | | | | |
| History of stroke/TIA | 2126 | 258 (21.4%) | 92 (10.0%) | **<0.001** |
| History of myocardial infarction | 2126 | 76 (6.3%) | 52 (5.7%) | 0.55 |
| Atrial fibrillation | 2126 | 315 (26.1%) | 8 (0.9%) | **<0.001** |
| Hypertension diagnosis | 2126 | 661 (54.7%) | 413 (45.0%) | **<0.001** |
| Hyperlipidemia diagnosis | 2126 | 195 (16.1%) | 146 (15.9%) | 0.88 |
| Diabetes diagnosis | 2126 | 181 (15.0%) | 129 (14.1%) | 0.55 |
| Depression diagnosis | 2126 | 130 (10.8%) | 87 (9.5%) | 0.33 |
| Anticoagulation treatment | 2126 | 330 (27.3%) | 23 (2.5%) | **<0.001** |
| **Neuroimaging** | | | | |
| Type of imaging  CT  MRI | 2060 | 881/1163 (75.8%)  282/1163 (24.2%) | 644/897 (71.8%)  253/897 (28.2%) | **0.04** |
| MTA rating, median [IQR] | 1744 | 2 [1–2] | 1 [1–2] | **0.03** |
| Fazekas rating, median [IQR] | 1737 | 1 [0.5–2] | 1 [0–2] | **0.001** |

Data are shown as mean (SD) and n (%), unless otherwise specified.

Abbreviations: CSF=cerebrospinal fluid; CT=computed tomography; FAS=verbal fluency test with letters F,A,S; IQR=interquartile range; MCI=mild cognitive impairment; MMSE=Mini-Mental State Examination; MRI=magnetic resonance imaging; MTA=medial temporal lobe atrophy; RAVLT=Rey Auditory Verbal Learning Test; RCF=Rey Complex Figure Test; SCI=subjective cognitive impairment; TIA=transient ischemic attack; WAIS-IV=Wechsler Adult Intelligence Scale, Fourth Edition.

**Supplementary Figure 1.** ATN biomarker profiles of patients in the MemClin cohort.

ATN profiles are shown for all patients with full data available for classification (n=774). Classification was based on CSF Aβ42 (marker of amyloid, A), CSF p-tau_181_ (marker of tau, T), and medial temporal lobe atrophy (MTA) (marker of neurodegeneration, N). Biomarker profiles are presented for the whole cohort (panel A) and for each diagnosis group (panels B-D). Abnormal CSF Aβ42 and p-tau_181_ were defined based on the standard method-specific cut-offs used in routine clinical practice. They were: Aβ42 ≤550 ng/l (ELISA), ≤599 ng/l (ECLIA), and ≤620 ng/l (CLEIA); p-tau ≥80 ng/l (ELISA), ≥56.5 ng/l (ECLIA), and ≥61 ng/l (CLEIA). Abnormal MTA was defined as ≥1.5 for patients <75 years, ≥2 for patients aged 75–84 years, and ≥2.5 for patients 85+ years.

Abbreviations: ATN=Amyloid-Tau-Neurodegeneration; Aβ=amyloid-β; CSF=cerebrospinal fluid; MTA=medial temporal lobe atrophy; p-tau=phosphorylated tau.

**Supplementary Figure 2.** Estimated eligibility for lecanemab among patients with MCI

Eligibility was determined according to the appropriate use recommendations (AUR) for lecanemab (Cummings et al., 2023). Abnormal CSF Aβ42 was considered sufficient evidence for AD pathology. Abnormal Aβ42 was defined based on the standard laboratory cut-offs used in routine clinical practice. The operationalization of the AUR criteria is described in detail in Supplementary material (Table 1); not all exclusion criteria could be assessed in our study, including MRI-related safety criteria (such as cerebral microbleeds, macrohemorrhage, lacunar infarcts, cortical superficial siderosis, and imaging findings suggestive of cerebral amyloid angiopathy).

Abbreviations: AUR=appropriate use recommendations; AD=Alzheimer’s disease; Aβ=amyloid-β; BMI=body mass index; CSF=cerebrospinal fluid; GDS=Geriatric Depression scale; HADS=Hospital Anxiety and Depression Scale; MCI=mild cognitive impairment; MMSE=Mini-Mental State Examination; MoCA=Montreal Cognitive Assessment; SCI=subjective cognitive impairment.

**Supplementary Figure 3.** Estimated eligibility for lecanemab among patients with dementia

Eligibility among patients with AD-type dementia: 23.3% (83/356). Eligibility was determined according to the appropriate use recommendations (AUR) for lecanemab (Cummings et al., 2023). Abnormal CSF Aβ42 was considered sufficient evidence for AD pathology. Abnormal Aβ42 was defined based on the standard laboratory cut-offs used in routine clinical practice. The operationalization of the AUR criteria is described in detail in Supplementary material (Table 1); not all exclusion criteria could be assessed in our study, including MRI-related safety criteria (such as cerebral microbleeds, macrohemorrhage, lacunar infarcts, cortical superficial siderosis, and imaging findings suggestive of cerebral amyloid angiopathy).

Abbreviations: AUR=appropriate use recommendations; AD=Alzheimer’s disease; Aβ=amyloid-β; BMI=body mass index; CSF=cerebrospinal fluid; GDS=Geriatric Depression scale; HADS=Hospital Anxiety and Depression Scale; MCI=mild cognitive impairment; MMSE=Mini-Mental State Examination; MoCA=Montreal Cognitive Assessment; SCI=subjective cognitive impairment.

**Supplementary Figure 4.** Estimated eligibility for donanemab among patients with MCI

Eligibility was determined according to the appropriate use recommendations (AUR) for donanemab (Rabinovici et al., 2025). Abnormal CSF Aβ42 was considered sufficient evidence for AD pathology. Abnormal Aβ42 was defined based on the standard laboratory cut-offs used in routine clinical practice. The operationalization of the AUR criteria is described in detail in Supplementary material (Table 2); not all exclusion criteria could be assessed in our study, including MRI-related safety criteria (such as cerebral microbleeds, macrohemorrhage, lacunar infarcts, cortical superficial siderosis, and imaging findings suggestive of cerebral amyloid angiopathy).

Abbreviations: AUR=appropriate use recommendations; AD=Alzheimer’s disease; Aβ=amyloid-β; CSF=cerebrospinal fluid; MCI=mild cognitive impairment; MMSE=Mini-Mental State Examination; MoCA=Montreal Cognitive Assessment; SCI=subjective cognitive impairment.

**Supplementary Figure 5.** Estimated eligibility for donanemab among patients with dementia

Eligibility among patients with AD-type dementia: 26.1% (93/356). Eligibility was determined according to the appropriate use recommendations (AUR) for donanemab (Rabinovici et al., 2025). Abnormal CSF Aβ42 was considered sufficient evidence for AD pathology. Abnormal Aβ42 was defined based on the standard laboratory cut-offs used in routine clinical practice. The operationalization of the AUR criteria is described in detail in Supplementary material (Table 2); not all exclusion criteria could be assessed in our study, including MRI-related safety criteria (such as cerebral microbleeds, macrohemorrhage, lacunar infarcts, cortical superficial siderosis, and imaging findings suggestive of cerebral amyloid angiopathy).

Abbreviations: AUR=appropriate use recommendations; AD=Alzheimer’s disease; Aβ=amyloid-β; CSF=cerebrospinal fluid; MCI=mild cognitive impairment; MMSE=Mini-Mental State Examination; MoCA=Montreal Cognitive Assessment; SCI=subjective cognitive impairment.

**Supplementary Figure 6.** Estimated eligibility for lecanemab (sensitivity analysis, assuming all patients without MRI to be ineligible)

Eligibility was determined according to the appropriate use recommendations (AUR) for lecanemab (Cummings et al., 2023). Abnormal CSF Aβ42 was considered sufficient evidence for AD pathology. Abnormal Aβ42 was defined based on the standard laboratory cut-offs used in routine clinical practice. The operationalization of the AUR criteria is described in detail in Supplementary material (Table 1); not all exclusion criteria could be assessed in our study, including MRI-related safety criteria (such as cerebral microbleeds, macrohemorrhage, lacunar infarcts, cortical superficial siderosis, and imaging findings suggestive of cerebral amyloid angiopathy).

Abbreviations: AUR=appropriate use recommendations; AD=Alzheimer’s disease; Aβ=amyloid-β; BMI=body mass index; CSF=cerebrospinal fluid; GDS=Geriatric Depression scale; HADS=Hospital Anxiety and Depression Scale; MCI=mild cognitive impairment; MMSE=Mini-Mental State Examination; MoCA=Montreal Cognitive Assessment; SCI=subjective cognitive impairment.

**Supplementary Figure 7.** Estimated eligibility for donanemab (sensitivity analysis, assuming all patients without MRI to be ineligible)

Eligibility was determined according to the appropriate use recommendations (AUR) for donanemab (Rabinovici et al., 2025). Abnormal CSF Aβ42 was considered sufficient evidence for AD pathology. Abnormal Aβ42 was defined based on the standard laboratory cut-offs used in routine clinical practice. The operationalization of the AUR criteria is described in detail in Supplementary material (Table 2); not all exclusion criteria could be assessed in our study, including MRI-related safety criteria (such as cerebral microbleeds, macrohemorrhage, lacunar infarcts, cortical superficial siderosis, and imaging findings suggestive of cerebral amyloid angiopathy).

Abbreviations: AUR=appropriate use recommendations; AD=Alzheimer’s disease; Aβ=amyloid-β; CSF=cerebrospinal fluid; MCI=mild cognitive impairment; MMSE=Mini-Mental State Examination; MoCA=Montreal Cognitive Assessment; SCI=subjective cognitive impairment.
